# Predictive value of gamma-glutamyl transpeptidase to lymphocyte count ratio in hepatocellular carcinoma patients with microvascular invasion

**DOI:** 10.1186/s12885-020-6628-7

**Published:** 2020-02-18

**Authors:** Hongxing Zhang, Yu Zhou, Yicheng Li, Wanying Qin, Yunhua Zi, Yulan Liu, Xiaoying Qiu, Hongyuan Xu, Weijia Liao, Zhaoquan Huang

**Affiliations:** 1 Laboratory of Hepatobiliary and Pancreatic Surgery, Affiliated Hospital of Guilin Medical University, Guilin, 541001 Guangxi People’s Republic of China; 2 Second clinical medical college, Guangxi Medical University, Nanning, 530021 Guangxi People’s Republic of China; 3 Department of Pathology, Guilin Medical University, Guilin, 541001 Guangxi People’s Republic of China

**Keywords:** Hepatocellular carcinoma, Microvascular invasion, GLR, Predictive

## Abstract

**Background:**

Microvascular invasion (MVI) is an independent risk factor for poor prognosis in hepatocellular carcinoma (HCC). However, there is still a lack of preoperative markers to predict MVI in HCC. This study intends to explore the potential application value of the gamma-glutamyl transpeptidase (GGT) to lymphocyte count ratio (GLR) in predicting MVI in HCC and provide guidance for clinical diagnosis and treatment.

**Methods:**

From March 2010 to December 2015, 230 HCC patients who underwent surgical treatment in the Affiliated Hospital of Guilin Medical University were selected. Clinicopathological parameters between the MVI group (*n* = 115) and the non-MVI group (n = 115) were comparatively analyzed. The GLR was used as the potential risk factor for HCC with MVI, and its optimal cut-off value was estimated by using the receiver operating characteristic (ROC) curve. The Kaplan-Meier method was used to analyze the survival of HCC patients, and univariate and multivariate Cox regression analyses were used to establish independent predictors affecting postoperative HCC patients.

**Results:**

The GLR levels in the MVI group and non-MVI group were 84.83 ± 61.84 and 38.42 ± 33.52 (*p* <  0.001), respectively. According to ROC curve analysis, the optimal cut-off value of GLR was 56.0, and the area under the ROC curve (AUC) was 0.781 (95% CI, 0.719–0.833) for the risk prediction of MVI in HCC patients. Multivariate analysis showed that tumor size > 5 cm, HCC combined with MVI and GLR >  56.0 were independent risk factors for poor prognosis in HCC patients. In addition, compared with the non-MVI group, patients in the MVI group had shorter progression-free survival (PFS) and overall survival (OS).

**Conclusion:**

GLR could be a predictive biomarker of HCC after operation and a potential predictor of HCC combined with MVI.

## Background

Hepatocellular carcinoma (HCC) is the most common primary liver malignancy. According to the global cancer statistics in 2018, the number of new cases of liver cancer reached 841,080, and there were 781,631 deaths [1]. Liver cancer is a significant public health problem worldwide, especially in China [2]. Studies have shown that MVI is a considerable risk factor for poor postoperative prognosis in HCC, MVI leads to early postoperative recurrence and metastasis and is an independent predictor of long-term postoperative survival [3, 4]. The number of MVI, depth of infiltration, and distance of invasion all affect the prognosis of postoperative HCC patients [5, 6]. In recent years, there have been many models for the diagnosis, treatment and prognosis of HCC [7, 8]. Therefore, the use of models to assess whether patients have preoperative HCC metastasis or HCC combined with MVI has tremendous clinical significance for selecting appropriate individualized treatment methods and improving the prognosis and survival of HCC patients.

Studies have shown that tumors are malignant transformations stimulated by long-term inflammatory factors, with inflammation as the driving factor and cancer as the result [9]. Inflammatory cytokines are essential participants in regulating the tumor microenvironment, and they can promote the proliferation and survival of tumor cells and enhance angiogenesis, invasion and metastasis [10]. Chronic inflammation and inflammation-related factors are the premise or basis of tumorigenesis. However, what are the vital inflammatory factors? There is no conclusion yet. Therefore, it is necessary to explore and identify the related inflammatory factors affecting the occurrence and progression of tumors to assist clinical diagnosis and treatment and achieve the fundamental purpose of the treatment or prevention of tumors. Recent studies have found that the GLR index plays an essential role in tumor progression, and its prognostic potential is superior to that of other inflammatory scoring systems [11]. It is speculated that GLR may be a critical factor in the occurrence of HCC combined with MVI and affect the survival and prognosis of HCC patients.

This study aims to explore the potential value of GLR in predicting the risk of HCC with MVI and its significance in predicting the prognosis of HCC to provide a new basis for the development of clinical treatment plans.

## Material and methods

### Patients

In this article, 230 patients with hepatocellular carcinoma (115 in the MVI group and 115 in the non-MVI group) who underwent surgical treatment at the Affiliated Hospital of Guilin Medical University were selected. All patients were diagnosed by clinical examination, ultrasonography (US), magnetic resonance imaging (MRI), thoracic and abdominal CT, angiography and hematology. The resected samples of all patients were confirmed by pathological examination. In this paper, according to the study by Sumie et al. [12], we defined the group in which MVI was not found as the non-MVI group, the group in which 1–5 MVIs was found as the M1 group, and the group in which more than 5 MVIs were found as the M2 group. Among the MVI patients, 70 patients were in the M1 group, and 45 patients were in the M2 group. Table 1 lists the clinicopathological parameters of the patients, such as demographic characteristics (age, sex, life history), history of hepatitis virus infection, hematological examination parameters (blood routine, liver function, protein levels, bilirubin, alpha-fetoprotein (AFP), GLR, etc.), and the characteristics of the tumor (the degree of cirrhosis, size and number). This study conformed to the Declaration of Helsinki and was approved by the research ethics committee of the Affiliated Hospital of Guilin Medical University. Written informed consent was obtained from all patients.

**Table 1:** Clinical and biochemical data of examined patients

Parameter	Non MVI group	MVI group	*p* value
	Mean ± SD* (*n* = 115)	Mean ± SD^a^ (n = 115)	
Sex: female / male (n)	21 / 94	9 / 106	**0.019**
Age (years)	50.23 ± 12.28	48.34 ± 11.00	0.221
Drinking: absent / present (n)	57 / 58	52 / 63	0.509
Smoking: absent / present (n)	73 / 42	66 / 49	0.345
HBsAg: negative / positive (n)	27 / 88	17 / 98	0.094
HCVAb: negative / positive (n)	109 / 6	113 / 2	0.280
Family history: absent/present (n)	99 / 16	95 / 20	0.468
Tumor number: single/multiple (n)	86 / 29	72 / 43	**0.047**
Tumor size (cm)	6.08 ± 3.32	8.53 ± 3.78	**< 0.001**
Liver cirrhosis: absent / present (n)	9 / 106	7 / 108	0.796
NEUT (× 10^9^/L)	3.71 ± 1.50	4.52 ± 2.24	**0.001**
LYMPH (× 10^9^/L)	1.77 ± 0.61	1.58 ± 0.59	**0.012**
WBC (× 10^9^/L)	6.17 ± 1.85	6.87 ± 2.50	**0.017**
Platelets (× 10^9^/L)	172.43 ± 72.73	180.14 ± 82.65	0.453
Albumin (g/L)	41.32 ± 4.01	39.47 ± 4.62	**0.002**
Globulin (g/L)	29.83 ± 4.79	31.34 ± 6.52	**0.045**
TBIL (μmol/L)	13.22 ± 4.93	15.07 ± 12.61	0.146
DBIL (μmol/L)	4.47 ± 2.32	5.48 ± 4.10	**0.022**
ALT (U/L)	37.38 ± 30.49	44.05 ± 33.32	0.115
AST (U/L)	39.33 ± 30.06	55.15 ± 35.29	**< 0.001**
ALP (U/L)	81.45 ± 87.65	102.22 ± 50.98	**0.030**
GGT (U/L)	59.17 ± 40.61	114.51 ± 64.46	**< 0.001**
AFP (ng/ml): median, range	44.60, 1.18–24,200	457.80, 0.61–25,410	0.271
GLR level	38.42 ± 33.52	84.83 ± 61.84	**< 0.001**

^a^Data presented as mean ± SD or proportions

*n* number of patients; *HBsAg* hepatitis B surface antigen, *HCVAb* hepatitis C virus antibody, *NEUT* neutrophil cell count, *LYMPH* lymphocyte count, *WBC* white blood cell, *TBIL* total bilirubin, *DBIL* direct bilirubin, *ALT* alanine aminotransferase, *AST* aspartate aminotransferase, *ALP* alkaline phosphatase, *GGT* gamma-glutamyl transpeptidase, *AFP* alpha-fetoprotein, *GLR* GGT to lymphocyte ratio

### Surveillance after hepatic resection

Based on the inclusion and exclusion criteria, a total of 230 patients with liver cancer who underwent radical resection entered the study. Patients were excluded if they had: (1) patients whose pathological diagnosis was not liver cancer (HCC), such as cholangiocarcinoma (CCC); (2) patients who died during the perioperative period; (3) patients with incomplete data or lost contact during the follow-up period; (4) infectious disease, immune system disease, blood system disease, or use of drugs that affect blood within 1 month; (5) patients underwent arterial chemoembolization before surgery or Radiofrequency ablation; (6) HIV positive patients. The contents and requirements of periodical follow-up, please refer to our previous and relevant reports [13, 14]. Progression-free survival (PFS) was defined as the period from the date of surgery to the date of metastasis, recurrence, death, or the last date on which the disease activity was evaluated, while overall survival (OS) was defined as the period from the date of surgery to the date of death or the last follow-up.

### Ascertainment of the cut-off value of GLR

To assess the risk of HCC combined with MVI, we analyzed the receiver operating characteristic (ROC) curve to determine the optimal cut-off value of preoperative GLR, which should have relatively high sensitivity and specificity. The other clinicopathological data were dichotomized: sex (male vs. female), age (> 50 age vs. ≤ 50 age), drinking (present vs. absent), HBsAg (positive vs. negative), tumor number (multiple vs. single), tumor size (> 5 cm vs. ≤ 5 cm), liver cirrhosis (present vs. absent), MVI type (MVI vs. non-MVI) and AFP level (> 20 ng/ml vs. ≤ 20 ng/ml).

### Statistical analysis

All statistical analyses were performed using SPSS 21.0 and MedCalc 11.3.0. For the count data conforming to a normal distribution, the independent sample t test was used for comparisons between the groups. For the data that did not conform to a normal distribution, a nonparametric test (Mann-Whitney U method) was used for group comparisons. The Pearson chi-square test was used to compare qualitative variables. The ROC curve and Youden index were used to select the optimal cut-off value of GLR; and the Kaplan-Meier method was used to obtain the survival curve. The log-rank test was used to study the differences among different groups. Then, the variables with *p* <  0.05 were analyzed by multivariate analysis. A Cox proportional hazards regression model was performed to determine the independent prognostic factors, and *p* <  0.05 was considered to indicate a significant difference.

## Results

### Correlation of MVI with the clinicopathological features of HCC patients, and GLR is more like an inflammatory factor

**Fig. 1:**
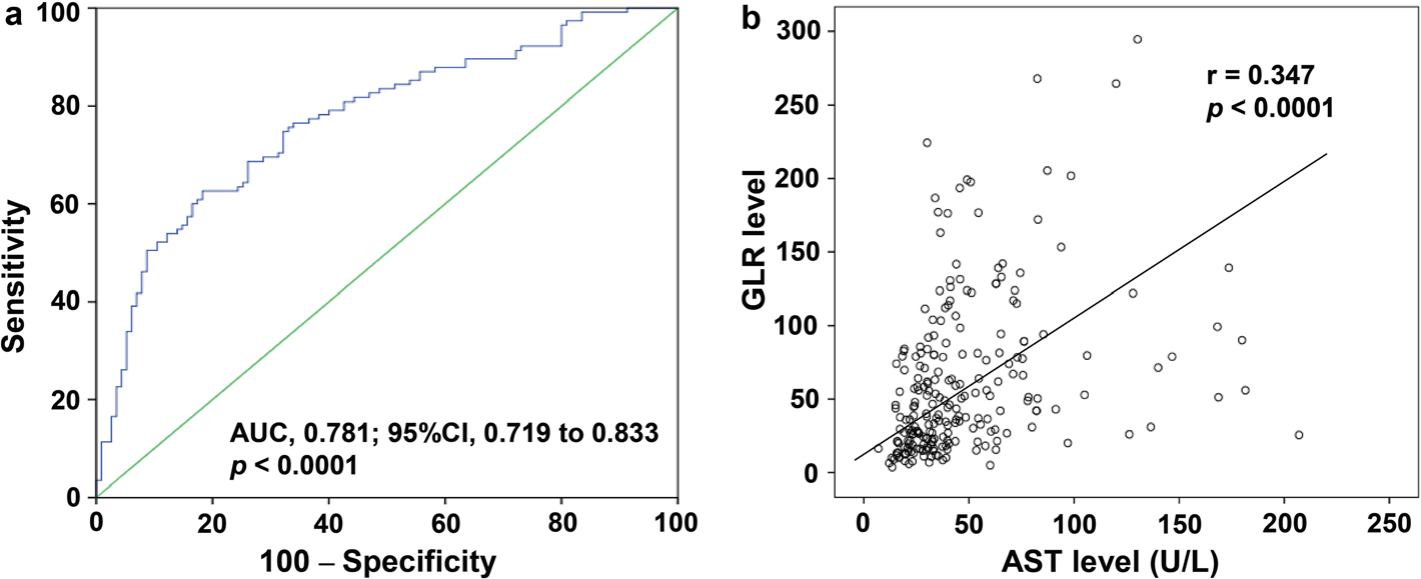
Determination of the optimal GLR cut-off value and its correlation analysis with AST. **a** The ROC curve of GLR in patients with HCC; **b** The positive relation between the GLR level and AST level. MVI, microvascular invasion; GLR, gamma-glutamyl transpeptidase to lymphocyte ratio; AST, aspartate aminotransferase; ROC, receiver operating characteristic

By comparative analysis of the clinicopathological parameters of the MVI group (*n* = 115) and non-MVI group (n = 115), it was found that the GLR levels in the MVI group and non-MVI group were 84.83 ± 61.84 and 38.42 ± 33.52, respectively (*p* <  0.001). In addition to the GLR level, the MVI group had larger tumor sizes and higher neutrophil cell count (NEUT), white blood cell (WBC), globulin, direct bilirubin (DBIL), aspartate aminotransferase (AST), alkaline phosphatase (ALP) and GGT levels (all *p* <  0.05) than the non-MVI group. In contrast, the lymphocyte count (LYMPH) and albumin levels were lower in the MVI group than in the non-MVI group (*p* <  0.05) (Table 1). In addition, we found that there was a positive correlation between the GLR level and AST level (r = 0.347, *p* <  0.001) (Fig. 1b). These results suggest that inflammatory factors, such as NEUT, AST and GLR etc., increase the risk of MVI in patients with HCC.

### GLR may be a potential predictor in HCC complicated with MVI

The ROC curve was drawn and analyzed according to the existence of MVI in patients with HCC. The optimum cut-off value of GLR was 56.0, the area under the ROC curve (AUC) was 0.781, and the 95% confidence interval (95% CI) was 0.719–0.833. The sensitivity and specificity were 63.6 and 81.7%, respectively, when the cut-off value of GLR was 56.0 (Fig. 1a). The results suggest that GLR may be a potential predictor for HCC complicated with MVI. Additionally, in the following studies, we found that when the cut-off value of GLR was 56.0, GLR had potential application value in predicting the postoperative survival of patients who had HCC and who had HCC combined with MVI.

### Univariate analysis and multivariate cox regression analysis indicated that GLR >  56.0 was an independent risk factor for postoperative HCC

In univariate analysis, GLR >  56.0 was found to be a risk factor for postoperative PFS (HR = 2.36, 95% CI, 1.53 - 3.08, *p* <  0.001) and OS (HR = 2.47, 95% CI, 1.80 - 3.40, *p* <  0.001). In addition to GLR >  56.0, the adverse factors of postoperative OS and PFS included multiple tumor nodules, tumor size > 5 cm, MVI and AFP >  20 ng/ml. The statistically significant factors in univariate analysis were further analyzed by the Cox proportional hazards regression model for multivariate analysis. GLR >  56.0 was an independent risk factor for postoperative PFS (HR = 1.56, 95% CI, 1.18 - 2.36, *p* = 0.017) and OS (HR = 1.63, 95% CI, 1.28 - 2.31, *p* = 0.006). In addition, tumor size > 5 cm and combined MVI can be used as independent risk factors for poor PFS and OS in HCC patients (Table 2).

**Table 2:** Analysis predictors of progression-free survival and overall survival in patients with HCC

Variable	Univariate analysis			Multivariate analysis		
	HR	95% CI	*p* value	HR	95% CI	*p* value
Progression-free survival						
Sex (male vs female)	1.27	0.73–2.37	0.507			
Age, y (> 50 vs ≤ 50)	1.16	0.78–1.72	0.446			
Drinking (present vs absent)	0.95	0.64–1.42	0.832			
HBsAg (positive vs negative)	0.82	0.49–1.37	0.469			
Tumor number (multiple vs single)	1.78	1.16–2.80	**0.007**			
Tumor size, cm (> 5 vs ≤ 5)	2.61	1.73–3.37	**< 0.001**	1.75	1.23–2.67	**0.003**
Liver cirrhosis (present vs absent)	1.33	0.65–2.75	0.429			
MVI (MVI vs non-MVI)	2.58	1.86–3.58	**< 0.001**	1.91	1.35–2.71	**< 0.001**
AFP, ng/ml (> 20 vs ≤ 20)	1.59	1.12–2.26	**0.010**			
GLR (> 56 vs ≤ 56)	2.36	1.53–3.08	**< 0.001**	1.56	1.18–2.36	**0.017**
Overall survival						
Sex (male vs female)	1.13	0.62–2.09	0.716			
Age, y (> 50 vs ≤ 50)	1.08	0.73–1.61	0.671			
Drinking (present vs absent)	0.83	0.56–1.24	0.371			
HBsAg (positive vs negative)	0.72	0.43–1.21	0.224			
Tumor number (multiple vs single)	1.83	1.20–2.66	**0.004**			
Tumor size, cm (> 5 vs ≤ 5)	2.87	2.10–3.76	**< 0.001**	2.11	1.48–3.07	**< 0.001**
Liver cirrhosis (present vs absent)	1.13	0.55–2.33	0.732			
MVI (MVI vs non-MVI)	2.83	2.05–3.61	**< 0.001**	2.00	1.41–2.84	**< 0.001**
AFP, ng/ml (> 20 vs ≤ 20)	1.64	1.15–2.33	**0.006**			
GLR (> 56 vs ≤ 56)	2.47	1.80–3.40	**< 0.001**	1.63	1.28–2.31	**0.006**

*HR* hazard ratio, *CI* confidence interval, *HBsAg* hepatitis B surface antigen, *MVI* microvascular invasion, *AFP* alpha-fetoprotein, *GLR* GGT to lymphocyte ratio

### The value of MVI and GLR in postoperative survival and prognosis of patients with HCC

**Fig. 2:**
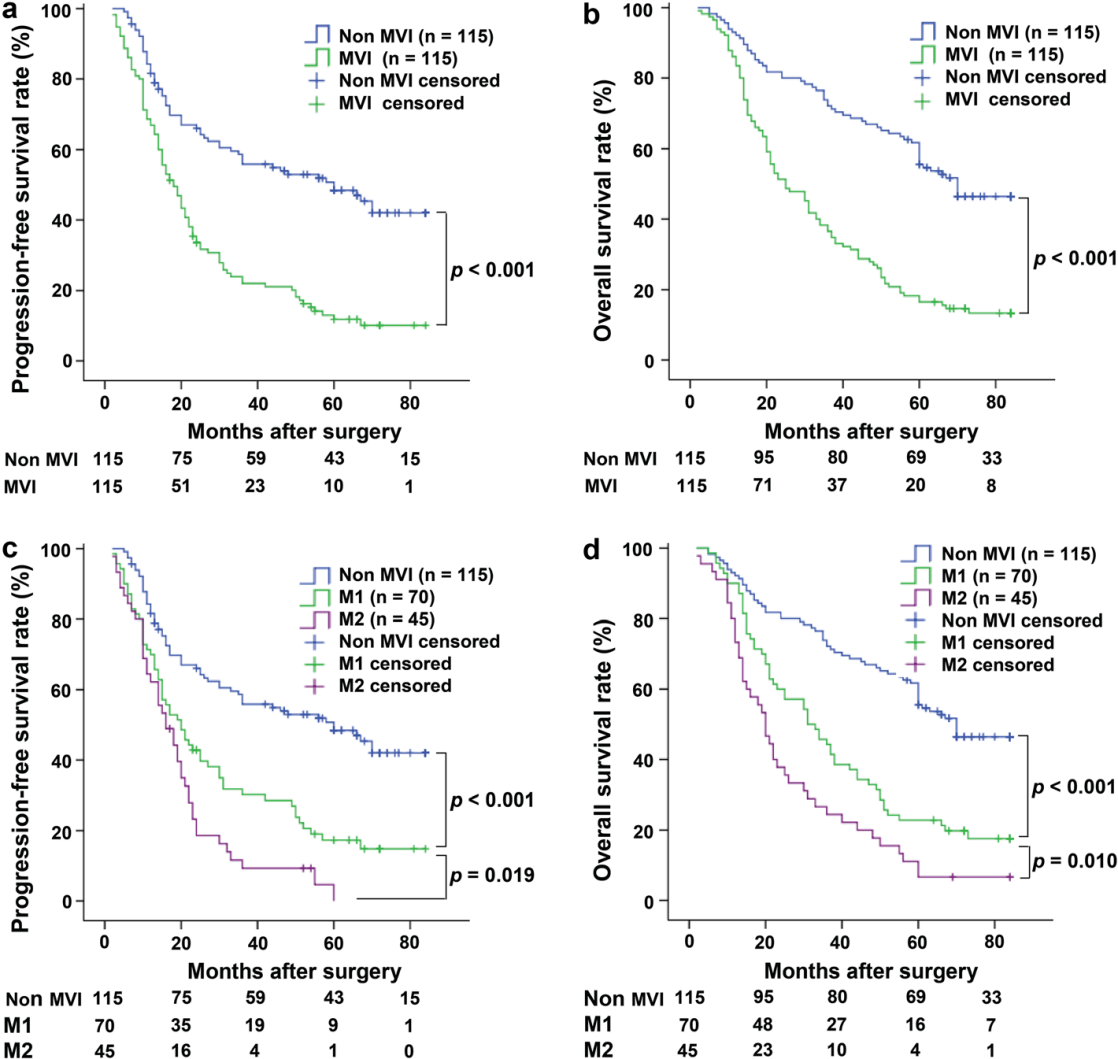
The survival rate was analyzed based on the presence of MVI in HCC patients. **a** Compared with the non-MVI group, the MVI group had significantly worse survival (*p* <  0.001). **b** Compared with the non-MVI group, the OS of the MVI group tended to be worse (*p* <  0.001). **c** The PFS of the non-MVI patients was significantly higher than that of the M1 patients (*p* <  0.001); the PFS of the M1 patients was significantly higher than that of the M2 patients (*p* = 0.019). **d** The OS of the non-MVI patients was significantly higher than that of the M1 patients (*p* <  0.001), and the OS of the M1 patients was significantly higher than that of the M2 patients (*p* = 0.010)

Kaplan-Meier analysis showed that the mean PFS and OS of the non-MVI group (*n* = 115) were 51.1 months and 59.3 months, and those of the MVI group (n = 115) were 26.9 months and 34.5 months, respectively. The PFS rates of the non-MVI group at 1 year, 3 years and 5 years were also significantly higher than those of the MVI group (73.6% vs. 59.4, 58.7% vs. 25.9 and 49.3% vs. 13.1%, respectively, all *p* <  0.001) (Fig. 2a). Similarly, the OS rates of the patients in the non-MVI group at 1 year, 3 years and 5 years were significantly higher than those of the MVI group (88.1% vs. 66.8, 73.0% vs. 37.1 and 61.4% vs. 17.9%, respectively, *p* <  0.001) (Fig. 2b). In addition, we further assessed the postoperative survival and prognosis of the M1 (*n* = 70) and M2 (*n* = 45) subgroups in the MVI group. Compared with the M2 group patients, the M1 group patients had a longer PFS (*p* = 0.019) (Fig. 2c) and OS (*p* = 0.010) (Fig. 2d).

**Fig. 3:**
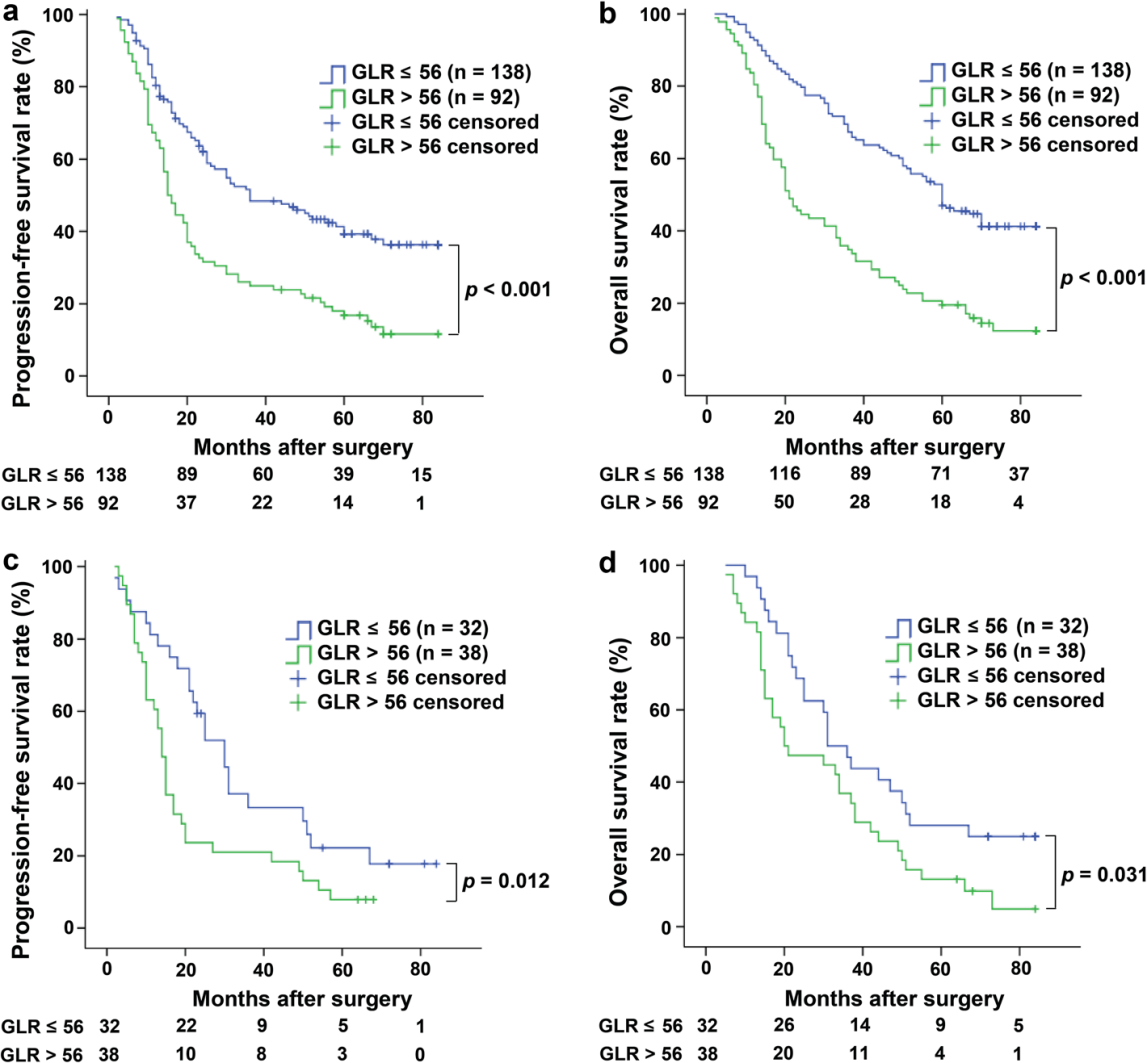
Kaplan-Meier analysis showed the relationship between GLR and the prognosis of HCC patients. The GLR ≤ 56.0 group (*n* = 138) had higher OS and PFS rates than the GLR > 56.0 group (*n* = 92) of HCC patients (both *p* <  0.001) (**a**, **b**). In the M1 group of patients (*n* = 70), the GLR ≤ 56.0 group (*n* = 32) had higher OS (*p* = 0.012) and PFS (*p* = 0.031) rates than the GLR > 56.0 group (*n* = 38) of HCC patients (**c**, **d**)

Of the 230 HCC patients involved in this study, compared with the GLR ≤ 56.0 group (*n* = 138), the GLR >  56.0 group (*n* = 92) had a shorter mean PFS (46.9 months vs 28.1 months, *p* <  0.001) and OS (55.8 months vs 33.4 months, *p* <  0.001) (Fig. 3a, b). More interestingly, in the M1 group, patients (n = 70) with GLR >  56.0 (*n* = 38) had a shorter mean PFS (36.7 months vs 21.5 months, *p* = 0.012) and mean OS (43.8 months vs 31.2 months, *p* = 0.031) (Fig. 3c, d). This finding indicates that GLR can also play a prognostic role in the M1 group of HCC patients.

These results suggest that MVI (MVI subgroup) and GLR level are closely related to postoperative survival and the prognosis of patients with HCC after operation.

## Discussion

The role of inflammatory factors in tumors has attracted much attention from researchers. Inflammation plays a decisive role in different stages of cancer development, including initiation, promotion, malignant transformation, invasion, and metastasis [15, 16]. In the early stage of tumorigenesis, inflammatory cells can become influential tumor promoters, creating a favorable environment for tumor growth and promoting blood vessel growth [17–19]. Many factors have been found to be related to the occurrence of MVI in HCC; in this study, it is suggested that GLR is positively correlated with AST, and NEUT, AST, GLR and other inflammatory factors’ level in the MVI group are higher than those in the non-MVI group, suggesting that these inflammatory factors may promote HCC progression and increase risk of MVI, which is in accordance with the researches which illuminate that anti-inflammatory treatment can effectively prevent early tumorigenesis and later malignant transformation [20, 21] .

In recent years, the construction of models for the diagnosis and prognosis prediction of hepatocellular carcinoma based on liquid biopsy has become a research hotspot [22–24]. The GLR prediction model built in this study was based on the ratio of GGT to lymphocyte count. Intrahepatic GGT mainly exists in the hepatocyte membrane and microsome and is a key enzyme in glutathione metabolism [25]; it plays an important role in hepatocarcinogenesis, vascular invasion and metastasis [26–28]. In addition, the overall survival rate of liver cancer patients with increased GGT is not favorable after liver tumor resection, radiofrequency ablation, and transcatheter arterial chemoembolization [29, 30]. Lymphocytes play a key role in cytotoxic cell apoptosis, inhibiting the production of inflammatory cytokines as well as the proliferation and migration of tumors in the body’s anti-tumor immune response [31–33]. In this study, the prediction of the postoperative survival of patients with HCC and HCC complicated with MVI by GLR was assessed, and the results showed that GLR can provide an excellent individualized prediction ability for HCC patients combined with MVI after operation. Univariate analysis of follow-up data showed that multiple tumor nodules, tumor size > 5 cm, MVI, AFP >  20 ng/ml and GLR >  56.0 were correlated with a shorter PFS and OS. Multivariate Cox regression analysis revealed that tumor size > 5 cm, MVI and GLR > 56.0 were independent predictors of poor prognosis for HCC after operation. This result is consistent with previous research findings that tumor size is an independent predictor of OS in HCC patients [34]. Other previous studies also showed that MVI is characterized by vascular infiltration and invasive phenotypes and is associated with the poor prognosis of liver cancer [35]. The results of univariate and multivariate Cox regression analyses of the subjects in this study were consistent with those of the above studies, which further indicates that HCC with tumor size > 5 cm and combined with MVI has a higher degree of malignancy.

This study further found that patients in the non-MVI group had a better OS and PFS than those in the MVI group, while the OS and PFS of the HCC patients in the M1 group were better than those in the M2 group. These results suggest that it is feasible and necessary for MVI patients to receive active surgical treatment. Although the univariate analysis used in this study showed that multiple tumor nodules and AFP >  20 ng/ml were predictors of undesirable PFS and OS, none of these factors were identified as independent predictors in the multivariate analysis. However, this does not mean that there is no association between these factors and HCC prognosis and metastasis; they can also be used as potential prognostic factors for HCC patients after resection. For example, multiple or single tumor nodules determine the prognosis of liver cancer patients treated differently [34], while a high level of AFP may affect the biological behavior of liver cancer, such as invasion, postoperative metastasis and recurrence, and prognosis. Compared with patients with AFP >  20 ng/ml, patients with AFP **≤** 20 ng/ml had a relatively better survival rate and prognosis [36].

## Conclusion

In clinical practice, the simple, reliable, low-cost and noninvasive serological indicators have significant value in the prediction of risk of MVI occurrence and postoperative prognosis for HCC patients. Elevated GLR level may result in MVI in liver cancer patients, which mechanism is complicated, thus, further researches to clarify it are in need. In conclusion, it is speculated that GLR may serve as an effective factor to predict the risk of MVI and prognosis of HCC patients. However, this is an retrospective study based on limited data from a single agency, and the selection bias cannot be avoided, thus, larger-scale and multi-center studies are in need, prospective studies are even better, in order to illuminate GLR’s underlying influence in liver cancer, and provide a basis for planning individual precise intervention therapy and improving prognosis for HCC patients.

## Data Availability

The datasets used and analyzed in the current study are available from the corresponding author upon reasonable request.

## Abbreviations

AFP: Alpha-fetoprotein
ALP: Alkaline phosphatase
ALT: Alanine aminotransferase
AST: Aspartate aminotransferase
AUC: Area under the ROC curve
CI: Confidence interval
DBIL: Direct bilirubin
GGT: Gamma-glutamyl transpeptidase
GLR: Gamma-glutamyl transpeptidase to lymphocyte count ratio
HBsAg: Hepatitis B surface antigen
HCC: Hepatocellular carcinoma
HR: Hazard ratio
LYMPH: Lymphocyte count
MVI: Microvascular invasion
NEUT: Neutrophil cell count
OS: Overall survival
PFS: Progression-free survival
ROC: Receiver operating characteristic
TBIL: Total bilirubin
WBC: White blood cell
